# First description of the karyotype and localization of major and minor ribosomal genes in *Rhoadsia altipinna* Fowler, 1911 (Characiformes, Characidae) from Ecuador

**DOI:** 10.3897/CompCytogen.v9i2.4504

**Published:** 2015-06-04

**Authors:** Omar Sánchez-Romero, César Quezada Abad, Patricio Quizhpe Cordero, Viviani França de Sene, Mauro Nirchio, Claudio Oliveira

**Affiliations:** 1 Universidad Técnica de Machala, El Oro, Ecuador; 2 Laboratório de Biologia e Genética de Peixes, Instituto de Biociências de Botucatu, Universidade Estadual Paulista (UNESP), Departamento de Morfologia, Distrito de Rubião Junior, Botucatu, São Paulo, Brazil. CEP: 18618-970; 3 Escuela de Ciencias Aplicadas del Mar, Universidad de Oriente, Estado Nueva Esparta, Venezuela; 4 Universidad Nacional Mayor de San Marcos UNMSM, Lima, Perú

**Keywords:** 18S and 5S ribosomal genes, C-bands, fishes, karyotype, NORs

## Abstract

Karyotypic features of *Rhoadsia altipinna* Fowler, 1911 from Ecuador were investigated by examining metaphase chromosomes through Giemsa staining, C-banding, Ag-NOR, and two-color-fluorescence in situ hybridization (FISH) for mapping of 18S and 5S ribosomal genes. The species exhibit a karyotype with 2n = 50, composed of 10 metacentric, 26 submetacentric and 14 subtelocentric elements, with a fundamental number FN=86 and is characterized by the presence of a larger metacentric pair (number 1), which is about 2/3 longer than the average length of the rest of the metacentric series. Sex chromosomes were not observed. Heterochromatin is identifiable on 44 chromosomes, distributed in paracentromeric position near the centromere. The first metacentric pair presents two well-defined heterochromatic blocks in paracentromeric position, near the centromere. Impregnation with silver nitrate showed a single pair of Ag-positive NORs localized at terminal regions of the short arms of the subtelocentric chromosome pair number 12. FISH assay confirmed these localization of NORs and revealed that minor rDNA clusters occur interstitially on the larger metacentric pair number 1. Comparison of results here reported with those available on other Characidae permit to hypothesize that the presence of a very large metacentric pair might represent a unique and derived condition that characterize one of four major lineages molecularly identified in this family.

## Introduction

The study of fish chromosomes has become an active area of research in recent decades providing basic information on the number, size and morphology of chromosomes, nucleolus organizers regions (NORs), distribution of constitutive heterochromatin and other more specific markers, detected through the application of molecular techniques (Nirchio and Oliveira 2006a). These features has been of great importance in allowing the diagnose of species, identification of differentiate cryptic species and chromosomal races (Nirchio et al. 2003a, 2005, 2007), establishing the relationships between species within a genus or family (Nirchio et al. 2001, 2006b, 2008, Oliveira et al. 2003), clarifying the origin of natural hybrids (Nirchio et al. 2003b) and increasing the knowledge of evolutionary mechanisms and genetic question in fishes (Nirchio et al. 2014).

Characiformes are exclusively freshwater fishes distributed in America and Africa, with the greatest diversity in major Neotropical watersheds (Buckup 1998). Characiformes comprises 2,081 valid species grouped in 23 families: Characidae is the largest with 15 subfamilies and 1,086 valid species (Eschmeyer and Fong 2015). These fish have the larger geographic distribution within this order occupying almost all environments of freshwater, with distribution in the Americas, from southwestern United States to South of Argentina (Lucena 1993). In Ecuador, among the freshwater fishes, the Characiformes is the second largest order for number of species (345), after Siluriformes (365) (Barriga 2012) and although chromosome studies in the Neotropical area have been performed for 475 species of Characiformes (Oliveira et al. 2009) until now there is an absolute absence of data from Ecuador.

**Figure 1.**
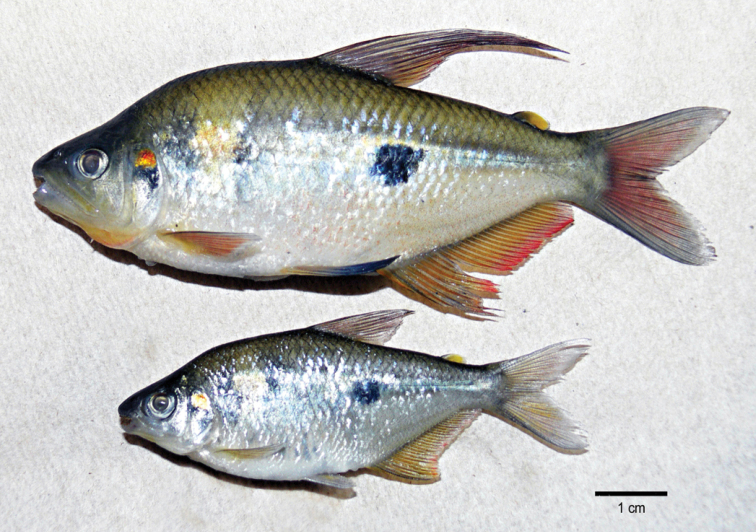
Male (**a**) and female (**b**) specimens of *Rhoadsia altipinna*.

The Rhoadsiinae, belonging to Characidae, includes three nominal genera: *Rhoadsia* with two species (*Rhoadsia altipinna*, *Rhoadsia minor* Eigenmann & Henn, 1914), *Parastremma* with three species (*Parastremma sadina* Eigenmann, 1912, *Parastremma album* Dahl, 1960, *Parastremma pulchrum* Dahl, 1960) and *Carlana* with only one species (*Carlana eigenmanni* (Meek, 1912)) (Cardoso 2003). In this work we present for the first time the cytogenetic description of *Rhoadsia altipinna* Fowler, 1911, which is characterized by a striking sexual dimorphism (Fig. 1). Species of *Rhoadsia* are distributed in Ecuador and Peru where they are relatively common and ecologically important. *Rhoadsia altipinna* occurs at low altitudes in the southwest region from the South of the Guayas River to North of the Peru, while *Rhoadsia minor* occurs at higher altitudes and in river systems in the Northwest of Ecuador (Barriga 2012). There are not cytogenetic data available for these species. The low diversity of species and peculiar geographical distribution of *Rhoadsia* species turn it in an interesting group from the evolutionary and conservation perspective, since in the western part of Ecuador, many areas within the range of the subfamily are under the condition of relatively serious threat (Loh et al. 2014).

## Methods

Twelve specimens of *Rhoadsia altipinna* (6 males and 6 females) were collected at Dos Bocas (03°16'07.6"S 079°44'14.8"W) in the Province El Oro, Ecuador were analyzed. Kidney cells suspensions were obtained from fishes injected intramuscularly with yeast glucose solution for mitosis stimulation 24 hours before injecting colchicine (Lee and Elder 1980). Chromosome preparations were obtained injecting 0.0125% colchicine intraperitoneally (0.5 ml/100 g body weight) 50 min before sacrificing as described by Nirchio and Oliveira (2006a). Following the guidelines of the American Veterinary Medical Association for euthanasia of animals (AVMA 2013), fish were sacrificed by numbing them with an overdose of Benzocaine (250 mg/L) until the cessation of opercula movement. Kidney were removed, homogenized and hypotonised by KCl 0,075 M for 20 min at 37 °C. Suspensions were centrifuged at 1000 rpm for 10 min. Supernatant was removed and the cells were fixed by cold fresh Carnoy (3:1 methanol and glacial acetic acid). This process was repeated three times and the cold fresh Carnoy was replaced after each centrifugation. Slides were prepared by conventional air draying method and stained for 20 min with 10% Giemsa in phosphate buffer, pH 6.88. No less than 10 metaphases per sample were analyzed both in males and females using separately all investigated techniques. Silver-stained nucleolus organizer regions (Ag-NORs) were obtained according to Howell and Black (1980). C-bands were obtained following the method of Sumner (1972).

Vouchers specimens were fixed in 10% formalin and deposited in the fish collection of the Laboratório de Biologia e Genética de Peixes (LBP), UNESP, Botucatu (São Paulo State, Brazil) (collection numbers LBP 19362), and Universidad Técnica de Machala (UTMach-020, 021, 047-052).

Position of major and minor ribosomal genes onto the chromosomes was mapped by fluorescence *in situ* hybridization (FISH), following the method of Pinkel et al. (1986). Major (18S rDNA) and minor (5S rDNA) ribosomal probes were isolated from the genome of *Moenkhausia sanctaefilomenae* (Steindachner, 1907) by PCR. Probe for rDNA was obtained using the primers 18S F (5’CCG CTT TGG TGA CTC TTG AT 3’) and 18S R (5’CCG AGG ACC TCA CTA AAC CA 3’) (White et al. 1990). This probe was labelled with Biotin-16-dUTP (Roche Applied Science) and hybridization signal detection of hybridization was performed using conjugated Avidin-Fluorescein (FITC). The 5S rDNA probe was obtained using the primer 5S F (5’TAC GCC CGA TCT CGT CCG ATC 3’) and 5S R (5’CAG GCT GGT ATG GCC GTA ACG 3’) (Pendás et al. 1994). This probe was labelled with Digoxigenin-11-dUTP (Roche Applied Science) and hybridization signal detection of hybridization was performed using Anti-Digoxigenin-Rhodamine (Roche Applied Science).

The mitotic figures were photographed using a Motic B410 microscope equipped with a Motic Moticam 5000C digital camera. Chromosomes were classified according to the arm ratio criteria (Levan et al. 1964). FISH metaphases were photographed with an Olympus BX61 photomicroscope equipped with a DP70 digital camera. Images were digitally processed with ADOBE PHOTOSHOP CS6 Extended.

## Results

**Figure 2.**
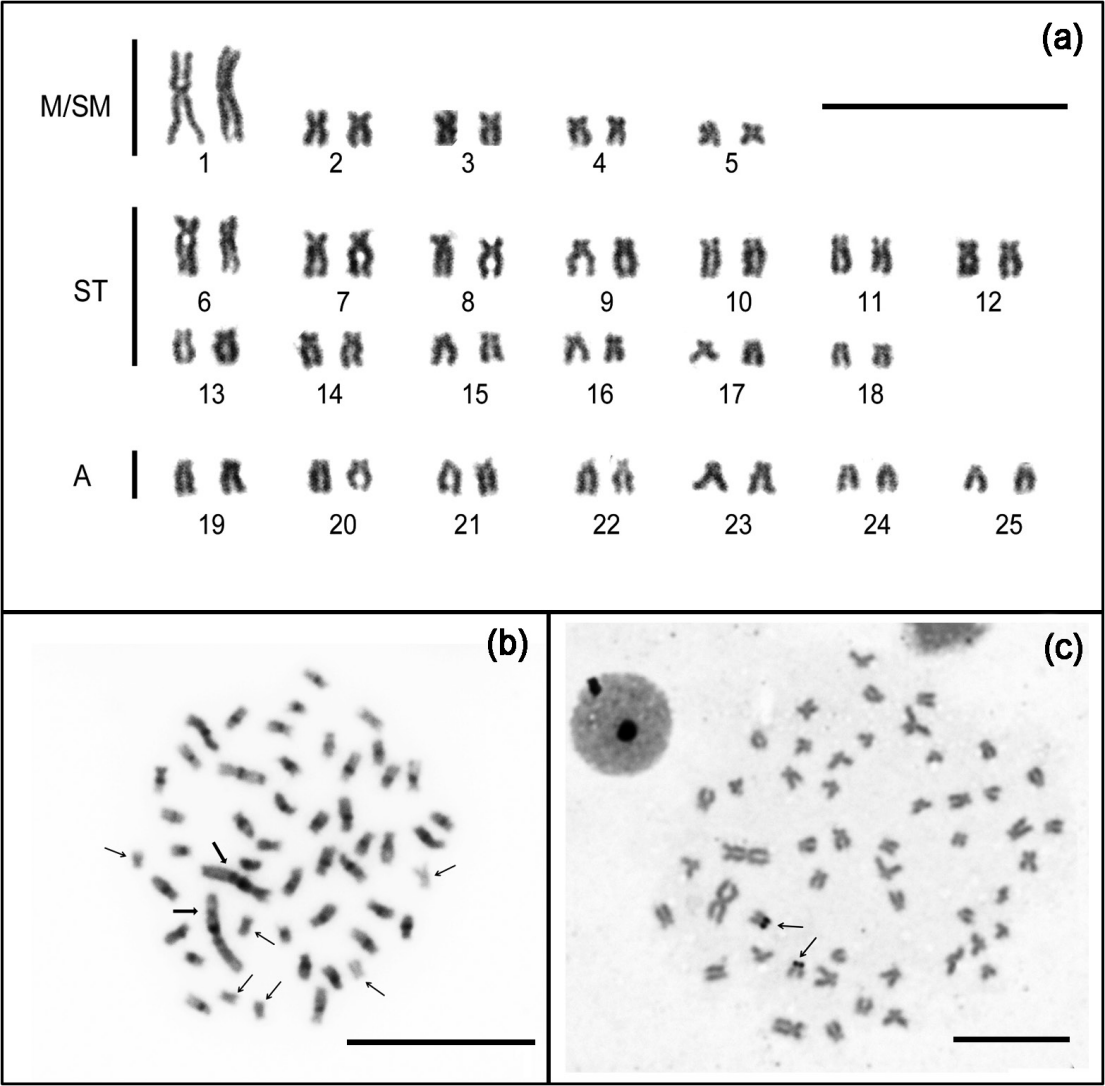
Chromosomes of *Rhoadsia altipinna* (male). (**a**) Giemsa-stained karyotype, M/SM: Metacentric/Submetacentric; ST: Subtelocentric; A: Acrocentric; (**b**) C-band somatic metaphases - thin arrows indicate chromosomes without positive C-bands and thick arrows point to heterochromatin on the pair number 1; (**c**) Silver-stained metaphase. Arrows indicate Ag-NORs. Bar =10 µm.

The analysis of 234 mitotic metaphase cells of *Rhoadsia altipinna* revealed a diploid number of 2n=50 chromosomes. The karyotype consisted of 10 metacentric, 26 submetacentric and 14 subtelocentric elements, with a fundamental number FN=86 (Fig. 2a). The larger metacentric pair (number 1), is about 2/3 longer than the average length of the rest of the metacentric series. No differences between chromosome complements were found.

Heterochromatin is distributed in paracentromeric position near the centromere of 44 chromosomes (Fig. 2b). The first metacentric pair presents two well-defined heterochromatic blocks in paracentromeric position, near to the centromere. Impregnation with silver nitrate (Fig. 2c) showed a single pair of Ag-positive NORs located at terminal regions of the short arms of the subtelocentric chromosome pair number twelve.

**Figure 3.**
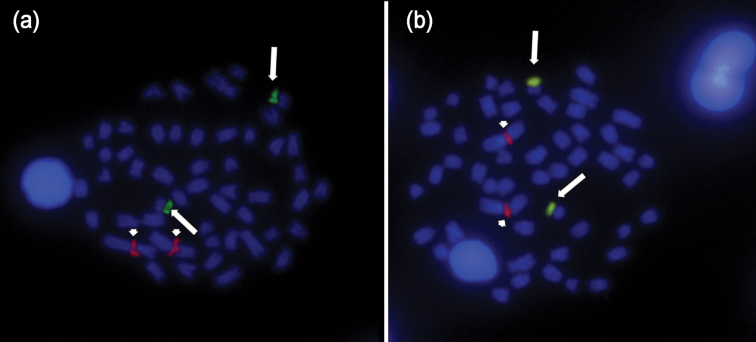
Dual Fluorescence *in situ* hybridization of 18S and 5S rDNA in male (**a**) and female (**b**) of *Rhoadsia altipinna*. Arrows point to hybridization signal of 18S rDNA, arrowheads indicate hybridization signal of 5S rDNA. Chromosomes are counterstained with DAPI.

Dual FISH with 18S and 5S rDNA probes (Fig. 3) confirmed the Ag-NOR sites and did not detect any further inactive major ribosomal clusters; in addition it showed that minor rDNA clusters occur interstitially on the larger metacentric pair number 1 and do not co-localize with the major rDNA clusters.

## Discussion

Cytogenetic studies in Characidae disclose great karyotype diversity related to the high variability of chromosome morphology among species and populations (Arai 2011), and the description of the karyotype of *Rhoadsia altipinna* adds new data to this picture. Indeed within the family although modal diploid number is relatively constant (2n= 50–52), FN is scattered over a wide range: from 56 in *Aphyocharax dentatus* Eigenmann & Kennedy, 1903 (Souza et al. 1995) to 132 in *Astyanax scabripinnis* (Jenyns, 1842) (Fauaz 1994). According to Arefjev (1994), the high morphological variability of karyotypes with simultaneous relatively constant diploid chromosome numbers is due to the occurrence of numerous chromosome inversions during the karyotype evolution in the group. A study performed from 1,135 living species contained in 12 families of the order Characiformes (Pazza and Kavalco 2010) revealed that Characidae are characterized by the highest rate of chromosomal changes.

Since this work reports the first description of the chromosome complement for *Rhoadsia altipinna* and karyotype description for its sister species, *Rhoadsia minor*, is not available yet, it is not possible to make more in-depth comparisons. Within the subfamily Rhoadsiinae, the karyotype of *Nematobrycon palmeri* Eigenmann, 1911 was published by Arefjev (1990) and, although the chromosomes are very condensed in his paper, their gross morphology is very similar to the observed here in *Rhoadsia altipinna*.

Dual FISH with 18S and 5S rDNA probes showed that in *Rhoadsia altipinna* minor ribosomal clusters occur interstitially on the larger metacentric pair number 1 and do not co-localize with the major rDNA clusters that are found in terminal position in an acrocentric pair. The presence of a single major rDNA cluster is the most common feature observed in fishes (Martins and Galetti 2001, Arai 2011). Although multiple 5S rDNA sites have been observed in a few species, such as *Astyanax scabripinnis* (Ferro et al. 2001) and *Hoplerythrinus unitaeniatus* (Spix & Agassiz, 1829) (Diniz and Bertollo 2003) the occurrence of single minor rDNA cluster close to centromeres is the most common feature in fish chromosomes (Martins and Galetti 2001, Mariguela et al. 2011) and it has been suggested that this position would be optimal for its organization in fish, since it has been recorder in most species of several orders (Martins and Wasko 2004).

In the more recent and comprehensive study on the phylogeny of the order Characiformes Oliveira et al. (2011) identified four major lineages in Characidae: (1) a clade composed by the single genus *Spintherobolus* Eigenmann, 1911 (without available cytogenetic information); (2) a clade named A, corresponding to Stevardiinae; (3) a clade named B composed by the subfamilies Tetragonopterinae, Characinae, Cheirodontinae, Aphyocharacinae and some small genera; (4) a clade named Clade C that includes also the subfamilies Rhoadsiinae, Stethaprioninae and many genera. Cytogenetic information is not available for *Spintherobolus* and in species of Clade A (Guimarães et al. 1995, Krinski et al. 2008, Pazian et al. 2012, Piscor et al. 2013) and Clade B (Martins-Santos and Tavares 1986, Souza et al. 1995, Alberdi and Fenocchio 1997, Mariguela et al. 2011) karyotypes do not show the big metacentric pair observed in *Rhoadsia altipinna*. On the contrary, all the Characidae species belonging to Clade C are characterized by the presence of the first large metacentric chromosome pair as shown by many reports on *Astyanax* Baird & Girard, 1854 (Carvalho et al. 2002), *Oligosarcus* Günther, 1864 (Shuhei et al. 2007), *Hollandichthys* Eigenmann, 1910 (Carvalho et al. 2002), *Hemigrammus* Gill, 1858 (Arefjev 1990), *Moenkhausia* Eigenmann, 1903 (Foresti et al. 1989), *Hyphessobrycon* Durbin, 1908 (Arefjev 1990, Carvalho et al. 2002, Mendes et al. 2011), among others. Thus the large metacentric chromosome pair seems to represent a unique and derived character of Clade C, which could reinforce its monophyly.
